# Do financial hardships affect health? A study among older adults in Switzerland

**DOI:** 10.1093/eurpub/ckad202

**Published:** 2023-11-23

**Authors:** Magali Dumontet, Yves Henchoz, Brigitte Santos-Eggimann

**Affiliations:** EconomiX, UPL, University Paris Nanterre, CNRS, Nanterre, France; Department of epidemiology and health systems, Centre for Primary Care and Public Health (Unisanté), University of Lausanne, Lausanne, Switzerland; Department of epidemiology and health systems, Centre for Primary Care and Public Health (Unisanté), University of Lausanne, Lausanne, Switzerland; Department of epidemiology and health systems, Centre for Primary Care and Public Health (Unisanté), University of Lausanne, Lausanne, Switzerland

## Abstract

**Background:**

A growing number of studies have underlined the relationship between socioeconomic status and health. Following that literature, we explore the causal effect of financial hardships on changes in health at older ages. Rather than traditional measures of socioeconomic variables, we study the role of financial hardships. The declarative measurement of financial hardships is particularly relevant for assessing the impact of short-term financial difficulties on health among older adults.

**Methods:**

In this study, we use data from the Lausanne cohort 65+. Participants are community-dwelling older adults representative of the population aged 65–70 years in 2004 and living in Lausanne (Switzerland) (*n* = 1352). We use longitudinal annual data with 11 years of follow-up (2006–16) to estimate dynamic panel models on several indicators measuring older adults’ health (self-rated health, number of medical conditions, depressive symptoms, difficulties with daily living activities).

**Results:**

We find evidence of causal effects of financial hardships on self-rated health (coef. = 0.059, *P* < 0.10) and on depressive symptoms (coef.=0.060, *P* < 0.05). On the other hand, we find no evidence of causality running from financial hardships to the number of medical conditions and the difficulties in daily living activities.

**Conclusion:**

These results make a contribution to the literature where nearly all previous research on associations between financial hardship and health does not establish causal relationships. Our results support the need to integrate health policies that mitigate the potential adverse health effects of financial hardship for older adults.

## Introduction

The positive correlation between health and socioeconomic status (SES) has been widely documented in economic, epidemiological and sociological studies.^1–4^ A wide variety of socioeconomic factors are associated with health across the life course. Researchers investigating health inequalities often acknowledge dynamic interdependencies in the relationship between SES and health.^5–6^ These studies use longitudinal data and attempt to understand the different causal effects whereby SES and health affect one another. Three mechanisms may underlie this positive association. First, SES affects health (social causation). For instance, scarce economic resources may lead to difficulties in securing medical care and delay in detecting conditions, reduced access to medical services (acute and preventive), or less effective treatment. Second, health affects SES (health selection). For instance, poor health may reduce the ability to work.^7^ Third, this association may also be explained by common factors such as genetic or childhood history.

In this study, we investigate the social causation pathway. Due to the reverse causality problem, previous studies on the social causation pathway have addressed this endogeneity problem by using dynamic panel data estimators,^8–11^ or by studying exogenous changes in income, e.g. lottery wins or income supplementation.^12–15^ Using six biennial waves of couples aged 51–61 in 1992 from the US Health and Retirement Study (HRS), Michaud and Van Soest find no causal effect of household wealth on health (measured with a multidimensional health index).^10^ On a sample of working age American individuals [Panel Study of Income Dynamics (PSID)], Halliday reports a causal effect of income on self-reported health for married women and men.^4^ Using more recent waves of PSID, Meraya et al. find evidence of a causal link from labor income to functional status.^11^ Using data from the British Household Panel Survey, Apouey and Clarck show that positive income shocks, measured by lottery winnings, have no significant effect on self-assed overall health, but a positive effect on mental health.^12^ Using an income supplementation experiment, Aguila et al. evaluate the health impact of additional income for poor older adults in the state of Yucatan in Mexico. They find strong evidence that income supplementation can have significant health benefit.^13^ In a similar vein, using the eligibility threshold of a Chilean basic pension program that grants a monthly payment to retirees without a contributory pension, Miglino et al. provide causal evidence that a permanent increase in older people’s income can improve their health.^14^ Inconsistent findings may in part reflect discrepancies in the target sample in terms of age, country and also in the measurement of economic status and health.

There has been an increasing interest in exploring the relationship between subjective SES and health.^15^^,^^16^ There are interdependencies between measures of SES and health.^5^^,^^6^^,^^17^ However, it has been argued that different demands for economic resources and different levels of assets can result in variations in household conditions. Traditional measures of socioeconomic variables may fail to capture this heterogeneity. Although researchers currently use actual income as a proxy for SES, financial hardship provides additional information about access to resources. People with similar incomes may experience wide variation in the financial strain they feel. The current study focuses on a specific measure of financial difficulties that is related to SES but does not capture it entirely. Financial hardship has been measured differently in various studies. Some studies have asked respondents whether there is any money left over at the end of month,^18^ others whether they suffer from financial hardship,^19^ and others whether they have difficulties meeting certain expenses.^20–22^ These studies indicate a positive association between financial hardship and health but do not measure a causal effect. Guan et al. report a systematic review of 40 observational studies quantifying the relationship between various measures of financial stress and depression outcomes in adults.^23^ A positive association between financial stress and depression is found. They conclude that more longitudinal research would be useful to investigate the causal relationship. Based on instrumental variable estimation, only Zimmerman and Katon find that financial strain is causally related to depression but that income is not.^24^

The measurement of financial hardship may be particularly useful for assessing the effect of economic resources on health in older adults. Occupation is not relevant due to retirement, and in their later years, some older adults may be exposed to major financial difficulties. Sirven et al. show that older adults with worsening economic conditions over time, simultaneously experience a rapid increase in their frailty symptoms.^25^ They find that a subjective measurement of deprivation (indicating whether the household is able to make ends meet) seems to better evaluate the household’s financial difficulties than an objective measure such as income. In a study linking material hardship to older adults’ health using a latent Markov model, Donni finds that individuals who previously experienced food insecurity or medication cutbacks are more likely to experience poorer health (measured by subjective health) in the subsequent time period.^21^

We contribute to the literature by investigating for the first time using panel dynamic models the causal effect of experiencing financial hardships on different dimensions of health for older adults.

## Methods

### Data and sample

Data were obtained from the first sample of the Lausanne cohort 65+ (Lc65+) that was launched in 2004 with a random selection of eligible individuals aged 65–70 in the community-dwelling population of Lausanne (a Swiss city of 125 000 inhabitants). The design is described in detail by Santos-Eggimann et al.^26^ After an enrollment questionnaire in 2004 and a baseline assessment in 2005 providing time-invariant variables, the same individuals are asked to complete a postal questionnaire every year. The Lc65+ study received approval from the Cantonal Human Research Ethical Committee (Protocol 19/04). Participants have been informed of the study goals and design and gave written consent to the study.

The study utilizes original longitudinal data for a period of 11 years (2006–16). Of the 1564 individuals enrolled in 2004, 1352 remained in 2006 as the first wave of the study. In terms of gender and birth year, the respondents are representative of the non-institutionalized inhabitants of the city of Lausanne. Suplementary A, Table 1 provides an overview of sample size, dropouts, and re-entrants by year. After 11 years of follow-up, 974 (72%) respondents to the first wave in 2006 were still alive and participated in 2016. A total of 926 respondents participated each year of the study period (2006–16). This is higher than the retention rates reported in other cohorts of community-dwelling older adults.^27–29^ As in all cohort studies, attrition rate was higher among individuals with unfavorable socioeconomic and health characteristics at recruitment. Henchoz et al. described attrition and show that dropping out was independent of sex, but positively associated with a foreign country of birth, a lower level of education, the presence of financial difficulties and a health status self-rated as average, poor or very poor.^30^

### Health status measurement

#### Self-rated health

Each respondent answers the question ‘How do you judge your current health’: very good, good, fair, poor, very poor. We code a variable bad self-rated health (SRH) as 1 if the respondent’s answer is poor or very poor and 0 otherwise.

#### Number of medical conditions

Lc65+ participants are asked about their medical conditions (‘During the past 12 months, have you been treated for, or suffered from, any of the following health problems as diagnosed by a physician’). In accordance with the list of medical diagnoses used in the Survey of Health, Ageing and Retirement in Europe (SHARE),^31^ only eight of these diagnoses are included in the variable ‘number of medical conditions’ (coronary heart disease, other heart diseases, stroke, diabetes mellitus, high blood pressure, chronic respiratory disease, arthritis and cancer).

#### Depressive symptoms

The presence of depressive symptoms was defined as a positive response to any of the two following questions of the Primary Care Evaluation of Mental Disorders Procedure: ‘During the past month, have you often been bothered by: (i) feeling down, depressed or hopeless? and (ii) little interest or pleasure in doing things?’. These two questions had a sensitivity of 96% and a specificity of 57% in diagnosing depression as compared with a standardized interview.^32^

#### Difficulties with daily living activities

Data about the basic and instrumental activities of daily living^33–34^ are collected by asking about having ‘no difficulties’, ‘reporting difficulties’, ‘help needed’ for nine basic and instrumental daily living activities (dressing, bathing, using the toilet, eating, getting in and out of the bed or armchair, using the telephone, shopping, handling finances, taking medication). Based on this information and using multiple correspondence analysis, we construct a score of difficulties with daily living activities. One dimension is retained from the analysis corresponding to 84.5% of principal inertia. The constructed score of difficulties in activities of daily living (called ‘DADL score’) is normalized to range between 0 and 1, with higher scores representing more severe difficulties with daily living activities.

### Measurement of financial difficulties

We use a declarative measurement of financial hardships. Participants are asked every year: ‘Have you been confronted with major financial difficulties in the last 12 months? Yes or No’. We consider that the respondent has been confronted with major financial difficulties if the respondent’s answer is yes.

### Control variables

The control variables are time variant factors: living alone and having a professional activity (‘None’, ‘Part-time professional activity’ (<15 h per week during the past 3 months) and ‘a regular professional activity’). We chose these variables because change in professional activity and change in living alone may be associated with change in health.^35^^,^^36^ Time-invariant factors such as education or gender are captured by first-differencing.

### Data analysis

We use unbalanced panel data where individual *i* is observed at time *t*. *h_it_* is the health variable for individual *i* at time *t*. First, we control for time-invariant unobserved heterogeneity by estimating first-difference models.

(1) $$\Delta h_{it}=\alpha \Delta {\mathrm{Financial}\;\mathrm{Difficulties}}_{it}+\beta \Delta X_{it}+\Delta \varepsilon_{it},$$

where $\Delta h_{it},\;\Delta {\mathrm{Financial}\;\mathrm{Difficulties}}_{it}$ and $\Delta X_{it}$ are the changes in health, in experiencing financial difficulties, and in the time-varying control variables, respectively, of individual *i* between time *t* and time *t−*1 and *ε_it_* is the error term. The first-difference estimator uses changes between two periods for each individual.

Second, we differentiate between negative and positive changes in health, as the effects of financial difficulties could be asymmetrical. Following the procedure developed by Meraya et al. and Mitra et al., we split the first-difference models by focusing separately on negative changes of health (health decline model: decline in SRH, increase of number of chronic conditions, appearance of depressive symptoms, increase in difficulties with daily living activities) and positive changes of health (health improvement model: improvement of SRH, decrease in the number of chronic conditions, disappearance of depressive symptoms, decrease in difficulties with activities of daily living).^11^^,^^37^ We also split changes in financial difficulties into (1) get out of financial difficulties and (2) appearance of financial difficulties.

We estimate a first-difference health decline model, where $\Delta h_{it}$ is a binary indicator variable measuring one-period change in health with the value of 1 representing health decline and zero representing no change or health improvement. $\Delta {\mathrm{Financial}\;\mathrm{Difficulties}}_{it}$ is a binary indicator variable measuring one-period change in financial difficulties with the value of 1 representing the appearance of financial difficulties and zero no change or the get out of financial difficulties.

Then, we estimate a first-difference health improvement model where $h_{it}$ is a binary indicator variable measuring one-period change in health with the value of 1 representing health improvement and zero representing no change or health decline. $\Delta {\mathrm{Financial}\;\mathrm{Difficulties}}_{it}$ is a binary indicator variable measuring one-period change in financial difficulties with the value of 1 representing the get out of financial difficulties and zero no change or the appearance of financial difficulties.

Third, we consider that current health is influenced by past health, by using the dynamic panel generalized method of moments (GMM) estimator. We report the one-step estimates of the first-difference GMM estimator.^9^ The proposed model for this estimator is:

(2) $$\Delta h_{it}=\beta_{1}\Delta h_{it-1}+\beta_{2}\Delta h_{it-2}+\beta_{3}\Delta h_{it-3}+\alpha \Delta {\mathrm{Financial}\;\mathrm{Difficulties}}_{it}+\beta_{4}{\Delta X}_{it}+\Delta \varepsilon_{it}.$$

This model also addresses the endogeneity problem caused by reverse causality.^38^ It tackles endogeneity problems (i) by instrumenting financial difficulties using its lagged value and (ii) by using first difference to deal with unobserved heterogeneity. This model also considers the endogeneity between current and past health. We estimate first-difference GMM models considering experiencing financial difficulties as a predetermined rather than an exogenous variable. It allows current values of $\varepsilon_{it}$ to be correlated with future values of financial difficulties. Based on the second-order auto-correlation test and the Hansen J statistics on overidentifying restrictions, we adjust for three lags of health measurement. For all specifications, we include time dummies to pick-up unobserved trends and time-varying explanatory variables (professional activity, living alone).

In a sensitivity analysis, we consider attrition bias by using sampling weights. Sampling weights were applied to keep participants at follow-up representative of the baseline sample in terms of birth country and education.

## Results

### Descriptive statistics

Table 1 describes the sample. For 2006, 59% of the sample are women and the average age is 70 years old. Nearly 36% of the sample live alone and 83% have no professional activity. For years 2008, 2011 and 2014, additional information on family income is available. In 2008, the family income averaged 5510 Swiss francs corresponding approximately to €3546 in 2008. In 2007, some 11% of respondents received supplemental retirement benefits, and 16% perceived subsidies for health insurance (information on supplemental retirement benefits and on subsidies for health insurance are available for 2004 and 2007).

**Table 1:** Descriptive statistics

	Total sample (*N* = 1352)	Financial difficulties in 2006 (*N* = 101)	No financial difficulties in 2006 (*N* = 1251)
Female (%)	59.1	58.4	59.2
Age (years, mean ± SD)	69.9 ± 1.4	69.9 ± 1.4	70.0 ± 1.4
Living alone (%)	36.3	45.5	35.6**
Education (%)			
No or mandatory school	25.6	33.6	24.9
Secondary education/apprenticeship	39.3	32.7	39.8
Baccalaureate or tertiary education	35.1	33.7	35.3
Professional activity (%)			
None	82.9	76.2	83.4
Part-time professional activity	9.1	12.8	8.8
Regular professional activity	7.9	10.9	7.7
Supplemental retirement benefits in 2007 (%)	10.5	43.0	7.9***
Subsidies for health insurance in 2007 (%)	15.3	55.7	12.0***
Income in 2008 (Swiss Francs, mean ± SD)	5509.1 ± 2678.7	3701.2 ± 2068.6	5630.4 ± 2672.1***

Notes: Descriptive statistics at t1 = 2006. Education assessed in 2004. χ^2^ proportion test, with H0: no difference by covariate in the proportion of respondents having financial difficulties.

*** Significant at 1%.

** Significant at 5%.

On average each year 6.7% of the respondents declare having financial difficulties. Despite an apparent stability of this proportion (Supplementary C, figure 2), the transition matrix indicates within variation; among those who declare having financial difficulties at *t *− 1, 48.9% declare that they do not have financial difficulties at wave *t*. Over the study period, 23.7% (*n* = 321) of respondents declare having financial difficulties at least once and 7.7% (*n* = 104) more than twice.

Individuals who declared having financial difficulties in 2006 were significantly more likely to live alone, to receive supplemental retirement benefits and subsidies for health insurance (table 1). The average income is lower among participants who declared having major financial difficulties.

Supplementary B, figure 1 and Supplementary C figure 2 summarize the change in health measures and the evolution of financial difficulties for respondents between 2006 and 2016. For the four variables, we observe deterioration in health.

### Econometric results

Table 2 summarizes the results of the first-difference models. For changes in financial difficulties, the coefficient is positive for all health measures, indicating that the transition into having financial difficulties (or getting out of financial difficulties) is associated with a change in health. The coefficient of change in financial difficulties is statistically significant for bad SRH (coef.=0.049, *P* < 0.05) and depressive symptoms (coef.=0.079, *P* < 0.01). Having a change in financial difficulties increases of 0.049 point the probability of having a change in SRH.

**Table 2:** Results of the first-difference models

	Bad self-rated health	Nb. medical conditions	Depressive symptoms	Difficulties in daily living activities
Living alone	−0.00533	0.0626	0.143***	−0.00468
	(0.0345)	(0.0469)	(0.0306)	(0.00314)
Reference: No professional activity				
Part-time professional activity	−0.0230	−0.000703	−0.0441**	−0.00423*
	(0.0215)	(0.0423)	(0.0224)	(0.00248)
Regular professional activity	0.0122	0.146*	0.0293	−0.00266
	(0.0306)	(0.0757)	(0.0339)	(0.00363)
Financial difficulties	0.0490∗∗	0.0723	0.0790***	0.00165
	(0.0206)	(0.0442)	(0.0214)	(0.00231)
Constant	0.0403∗∗∗	0.0505∗∗∗	0.0125***	0.00324∗∗∗
	(0.0142)	(0.00833)	(0.00443)	(0.000543)
Time dummies	Yes	Yes	Yes	Yes
Observations	10 918	10 791	10 736	10 691

Note: Standard errors are in parentheses.

* *P* < 0.10.

** *P* < 0.05.

*** *P* < 0.01.

Results from the split first-difference model that differentiates between negative and positive changes in health are provided in table 3. Table 3A indicates that the appearance of financial difficulties is significantly associated with a deterioration of health for all health variables. Table 3B indicates that getting out of financial difficulties is significantly associated with a health improvement for all health variables.

**Table 3:** Health decline and health improvement models

A) Health decline models	Self-rated health decline	Increase in number of chronic conditions	Appearance of depressive symptoms	Increase in difficulties with daily living activities
Living alone	0.0000370	0.0272	0.118***	−0.0289
	[−0.0475 to 0.0476]	[−0.0170 to 0.0714]	[0.0729 to 0.163]	[−0.0689 to 0.0111]
Reference: No professional activity				
Part-time professional activity	−0.0137	−0.0183	−0.0180	−0.00856
	[−0.0426 to 0.0152]	[−0.0584 to 0.0218]	[−0.0499 to 0.0138]	[−0.0406 to 0.0234]
Regular professional activity	0.00413	0.00752	0.0181	−0.0664**
	[−0.0352 to 0.0434]	[−0.0616 to 0.0766]	[−0.0227 to 0.0589]	[−0.131 to −0.00213]
Experiencing financial difficulties	0.0475**	0.0876***	0.0969***	0.0877***
	[0.00994 to 0.0850]	[0.0366 to 0.139]	[0.0534 to 0.140]	[0.0362 to 0.139]
Constant	0.109***	0.184***	0.113***	0.198***
	[0.0889 to 0.130]	[0.159 to 0.209]	[0.0921 to 0.133]	[0.172 to 0.223]
Time dummies	Yes	Yes	Yes	Yes
*N*	10 918	10 791	10 736	10 791

**B) Health improvement models**	**Improvement of self-rated health**	**Decrease in number of chronic conditions**	**Disappearance of depressive symptoms**	**Decrease in difficulties with daily living activities**

Living alone	0.00524	−0.0236	−0.0235	0.0350^*^
	[−0.0271 to 0.0375]	[−0.0691 to 0.0219]	[−0.0549 to 0.00796]	[−0.00497 to 0.0749]
Reference: No professional activity				
Part-time professional activity	0.00925	−0.0240	0.0250**	0.0209
	[−0.0135 to 0.0320]	[−0.0607 to 0.0128]	[0.00238 to 0.0476]	[−0.00426 to 0.0460]
Regular professional activity	−0.00809	−0.0720**	−0.0143	−0.00751
	[−0.0471 to 0.0309]	[−0.136 to −0.00810]	[−0.0588 to 0.0302]	[−0.0577 to 0.0426]
Getting out financial difficulties	0.0409**	0.0881***	0.0756***	0.0701***
	[0.00625 to 0.0756]	[0.0399 to 0.136]	[0.0377 to 0.113]	[0.0254 to 0.115]
Constant	0.0693***	0.214***	0.0773***	0.155***
	[0.0525 to 0.0861]	[0.188 to 0.241]	[0.0602 to 0.0945]	[0.131 to 0.178]
Time dummies	Yes	Yes	Yes	Yes
*N*	10 918	10 791	10 736	10 691

Note: Confidence intervals at 95% are in brackets.

* *P *<* *0.10.

** *P *<* *0.05.

*** *P *<* *0.01.

In table 4, first-difference GMM models indicate a causal effect of experiencing financial difficulties on SRH (coef.=0.059, *P* < 0.10), and on depressive symptoms (coef.=0.0854, *P* < 0.01). However, we find no evidence of causality running from financial hardships to the number of medical conditions and on difficulties in daily living activities.

**Table 4:** Results for the first-difference GMM models with financial difficulties variable considered as predetermined

	Bad self-rated health	Nb. medical conditions	Depressive symptoms	Difficulties in daily living activities
*ht *−* *1	0.187∗∗∗	0.0986∗∗	0.129***	0.224
	(0.0359)	(0.0410)	(0.0347)	(0.208)
*ht *−* *2	0.107∗∗∗	0.0418	0.0304	0.0561
	(0.0267)	(0.0304)	(0.0260)	(0.0923)
*ht *−* *3	0.0440∗∗	0.0118	−0.00947	0.0814
	(0.0215)	(0.0210)	(0.0217)	(0.0662)
Financial difficulties	0.0593*	0.0977	0.0854***	0.00262
	(0.0325)	(0.0666)	(0.0326)	(0.00356)
Living alone	0.0261	0.0473	0.186***	−0.00336
	(0.0423)	(0.0582)	(0.0392)	(0.00336)
Reference: No professional activity				
Part-time professional activity	−0.00456	−0.0220	−0.0511	−0.000635
	(0.0329)	(0.0584)	(0.0321)	(0.00231)
Regular professional activity	−0.0356	0.184	0.0461	0.00426
	(0.0524)	(0.130)	(0.0396)	(0.00420)
Time dummies	Yes	Yes	Yes	Yes
Observations	7006	6806	6755	6507
Individuals	1182	1179	1169	1176
N. instruments	75	77	75	77
AR2	−0.267	1.290	0.267	0.286
	*P*=0.790	*P*=0.197	*P*=0.790	*P*=0.775
Hansen J	59.38	69.96	56.73	59.84
	*P*=0.535	*P*=0.256	*P*=0.631	*P*=0.590

Note: Standard errors are in parentheses.

* *P* < 0.10.

∗∗ *P* < 0.05.

∗∗∗ *P* < 0.01.

### Sensitivity analysis

Results shown in tables 2–4 remain almost unchanged when using sampling weights to keep participants at follow-up representative of the baseline sample (Supplementary D).

## Discussion

Using original data from the Lausanne cohort 65+ with 11 years of follow-up, we investigate the short-term causal effect of experiencing financial hardships on health for older adults. This article provides two main contributions to the literature.

First, we contribute to the literature by measuring a causal effect where nearly all previous research on associations between subjective SES and health was conducted in a non-causal framework. Our methodology addresses the endogeneity problem caused by reverse causality between health and financial difficulties. We use first-difference GMM models and measure for the first time the causal effect of experiencing financial hardships on different indicators of health for older adults. We show that financial difficulties have a causal effect on SRH and depressive symptoms for older adults. However, this causal effect is not significant for the other health indicators: number of medical conditions and difficulties with daily living activities. In this paper, we examine short-term effects of experiencing financial difficulties. This time frame may explain why financial difficulties affect self-rated measures such as SRH and mental health but has no effect on physical function and medical conditions. In this regard, future work may develop alternative approaches to better understand the role of multiple episodes of financial difficulties on health over the life course.

Second, we make a contribution to the literature by addressing the relationship between experiencing financial hardships and health for older adults. Our results are in line with previous studies^21^^,^^25^ showing that subjective measures of deprivation are particularly relevant for older adults. As a proxy of SES, financial hardships provide additional information about access to resources. During the period, nearly 23.7% of the individuals declared experiencing financial difficulties at least once. They are poorer than others with a lower average income. They also benefit more from social benefits (supplemental retirement benefits and subsidies for health insurance). However, our results support the need to integrate for older adults, health policies that mitigate the potential adverse effects of experiencing financial difficulties on health and more particularly for mental health.

Several limitations should be mentioned. First, we use financial difficulties as a proxy for socioeconomic position. Even if individuals experiencing financial difficulties are poorer than others, we cannot disentangle individuals who declare financial difficulties because they have difficulties buying basic goods from individuals who have difficulties buying unnecessary goods. Second, small effect sizes may limit the public health significance of the results. Nevertheless, considering the multiple factors underlying complex constructs such as health perception and depression, even small causal effects appear meaningful.^39^ Finally, though we employed statistical techniques to address endogeneity from reverse causality and omitted variables, we cannot rule out the possibility that results may be affected by events not taken into account, such as the death of a child or moving house.

In conclusion, this study provides evidence of a causal pathway linking financial hardships to depression and poor health among older adults. Even in a high-income country like Switzerland, public policies should address social inequalities to promote healthy ageing. Further research should focus on better understanding the underlying mechanisms, which may not be the same if financial hardship is driven by difficulties getting basic necessities or less essential goods.

## Supplementary Material

ckad202_Supplementary_Data

## Data Availability

The data underlying this article will be shared on reasonable request to the corresponding author or the principal investigator of the Lc65+ study. Key points Declarative measurement of financial hardships is particularly relevant for assessing the impact of short-term financial difficulties on health among older adults. This study provides evidence of a causal pathway linking financial hardships to depressive symptoms and self-rated health among older adults. We find no evidence of causality running from financial hardships to the number of medical conditions and the difficulties in daily living activities. Even in a high-income country like Switzerland, public policies should address social inequalities to promote healthy aging.
