# Supplementary material for: Do financial hardships affect health? A study among older adults in Switzerland
Source: Eur J Public Health. 2023 Nov 23;34(1):7–13. doi: 10.1093/eurpub/ckad202 (PMC10843939; doi:10.1093/eurpub/ckad202)
Supplement: ckad202_Supplementary_Data [file ckad202_supplementary_data.pdf]

Supplementary material

A). Description of the sample

A. Table 1: Sample size, dropouts and estimation sample by year

Years (t)	Sample size	Dropouts	Dead	Re-entrants
2006	1352			
2007	1315	37	12	12
2008	1273	34	16	8
2009	1223	43	13	6
2010	1199	21	15	12
2011	1167	25	17	10
2012	1093	64	18	8
2013	1096	10	14	27
2014	1054	29	17	4
2015	1021	19	28	14
2016	974	27	28	8

$$Samplesize_t = Samplesize_{t-1} - Dropouts_t - Dead_t + Re - entrants_t$$

Re-entrants for a given year are participants who responded that year but not the previous year.

The number of deaths in the cohort corresponds to the sum of the numbers in column 4 (n=178).

B) Evolution of health variables

Figure 1: Average annual health variables

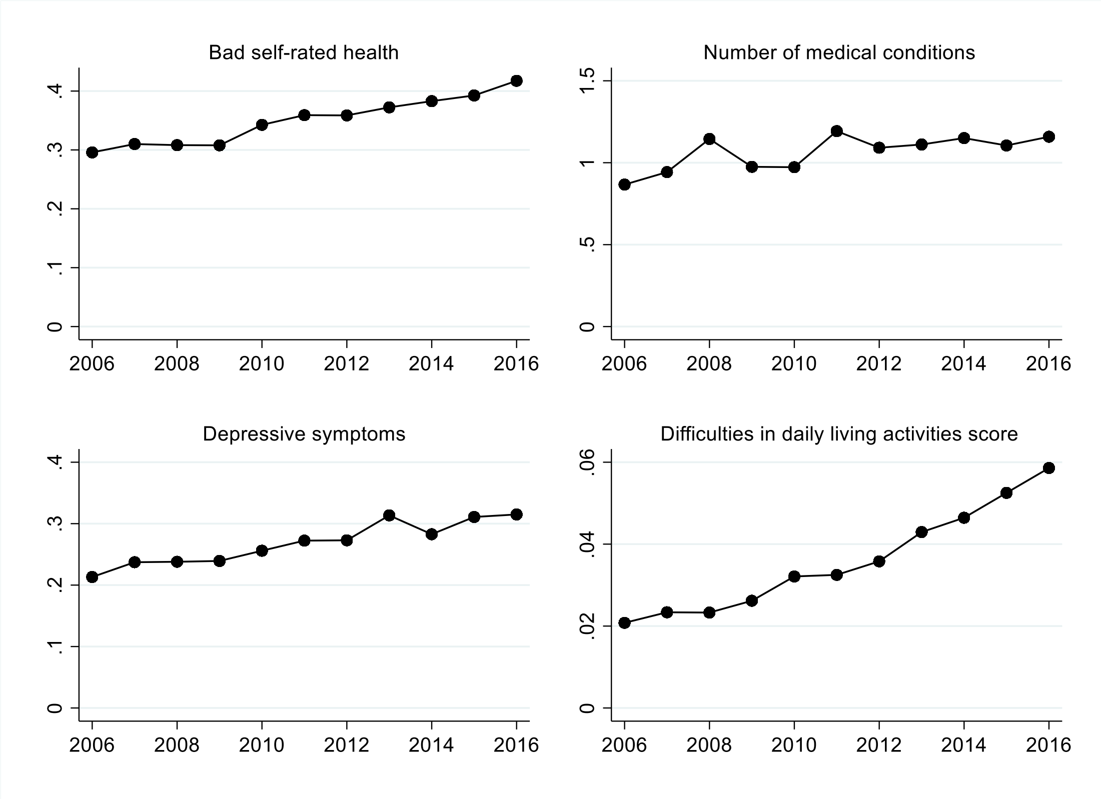

Legend : In 2010, nearly 30% of respondents declare a poor or very poor health. In 2010, respondents declare on average suffering from one medical conditions, 7% have depressive symptoms and the average score of Difficulties in daily living activities is nearly 0.03.

C) Evolution of financial difficulties

Figure 2: Average major financial difficulties by year

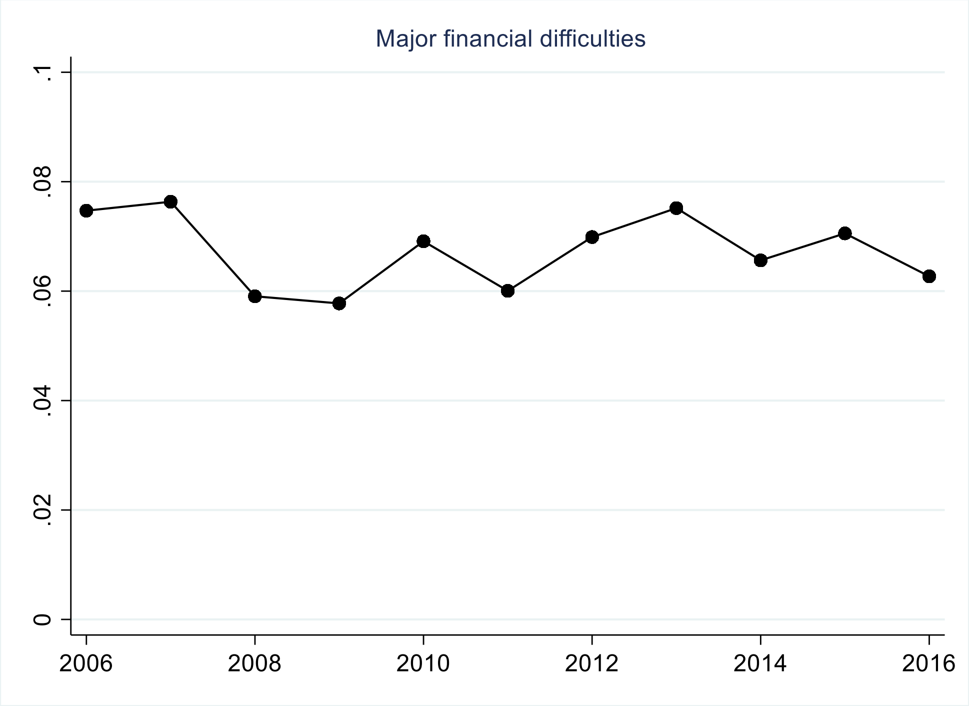

Legend: In 2011, 6% of the respondents declare having financial difficulties.

D) Sensitivity analysis : Sampling weights

Table D-2: Results of the first-difference models: sampling weights applied in Table 2

	**Bad self-rated health**	**Nb. medical conditions**	**Depressive symptoms**	**Difficulties in daily living activities**
Living alone	-0.0173	0.0771	0.140***	-0.00452
	(0.0366)	(0.0492)	(0.0301)	(0.00347)
Reference: No professional activity				
Part-time professional activity	-0.0199	0.0199	-0.0439+	-0.00471
	(0.0221)	(0.0434)	(0.0245)	(0.00300)
Regular professional activity	0.0109	0.162**	0.0380	-0.00257
	(0.0308)	(0.0771)	(0.0387)	(0.00376)
Financial difficulties	0.0530**	0.0856+	0.0795***	0.00210
	(0.0206)	(0.0451)	(0.0223)	(0.00287)
Constant	0.0409***	0.0499***	0.0119**	0.00340***
	(0.0140)	(0.00859)	(0.00461)	(0.000608)
Time dummies	Yes	Yes	Yes	Yes
Observations	10918	10791	10736	10691

Standard errors in parentheses
* p < 0.10,** p < 0.05, ***p < 0.01

Table D-3 : Health decline and health improvement models: sampling weights applied in Table 3

**A)Health decline models**				
	Self-rated health decline	Increase in number of chronic conditions	Appearance of depressive symptoms	Increase in difficulties with daily living activities
Living alone	-0.00443	0.0319	0.118***	-0.0299
	[-0.0527,0.0439]	[-0.0134,0.0772]	[0.0731,0.163]	[-0.0702,0.0104]
Reference: No professional activity				
Part-time professional activity	-0.0112	-0.00920	-0.0187	-0.00606
	[-0.0414,0.0189]	[-0.0501,0.0317]	[-0.0512,0.0137]	[-0.0394,0.0272]
Regular professional activity	0.00787	0.0116	0.0154	-0.0650+
	[-0.0298,0.0456]	[-0.0576,0.0809]	[-0.0296,0.0603]	[-0.131,0.000760]
Experiencing financial difficulties	0.0496**	0.0935***	0.0933***	0.0851***
	[0.0116,0.0877]	[0.0424,0.145]	[0.0489,0.138]	[0.0328,0.137]
Constant	0.108***	0.228***	0.113***	0.222***
	[0.0873,0.128]	[0.200,0.255]	[0.0925,0.134]	[0.194,0.250]
Time dummies	Yes	Yes	Yes	Yes
N	10918	10791	10736	10791

**B)Health improvement models**				
	Improvement of self-rated health	Decrease in number of chronic conditions	Disappearance of depressive symptoms	Decrease in difficulties with daily living activities
Living alone	0.0127	-0.0294	-0.0217	0.0381+
	[-0.0234,0.0487]	[-0.0770,0.0183]	[-0.0538,0.0103]	[-0.00397,0.0802]
Reference: No professional activity				
Part-time professional activity	0.00854	-0.0276	0.0242+	0.0185
	[-0.0144,0.0315]	[-0.0654,0.0102]	[-0.00226,0.0507]	[-0.00729,0.0443]
Regular professional activity	-0.00306	-0.0768**	-0.0256	-0.00604
	[-0.0446,0.0384]	[-0.142,-0.0118]	[-0.0766,0.0254]	[-0.0585,0.0464]
Getting out financial difficulties	0.0416**	0.0924***	0.0818***	0.0696***
	[0.00647,0.0767]	[0.0428,0.142]	[0.0419,0.122]	[0.0234,0.116]
Constant	0.0671***	0.188***	0.0792***	0.158***
	[0.0507,0.0835]	[0.162,0.214]	[0.0614,0.0971]	[0.133,0.182]
Time dummies	Yes	Yes	Yes	Yes
N	10918	10791	10736	10691

Confidence intervals at 95% are in brackets.

$^{+}$ $p < 0.10$, ** $p < 0.05$, *** $p < 0.01$

Table D.4: Results for the first-difference GMM models with financial difficulties variable considered as predetermined: sampling weights applied in Table 4

	**Bad self-rated health**	**Nb. medical conditions**	**Depressive symptoms**	**Difficulties in daily living activities**
h_{t-1}	0.185***	0.0989**	0.134***	0.197
	(0.0360)	(0.0414)	(0.0353)	(0.200)
h_{t-2}	0.107***	0.0442	0.0293	0.0506
	(0.0272)	(0.0331)	(0.0259)	(0.0908)
h_{t-3}	0.0422^{+}	0.00792	-0.0151	0.0679
	(0.0216)	(0.0213)	(0.0217)	(0.0663)
Financial difficulties	0.0644**	0.120^{+}	0.0915***	0.00196
	(0.0321)	(0.0674)	(0.0331)	(0.00363)
Living alone	0.0144	0.0617	0.181***	-0.00427
	(0.0443)	(0.0594)	(0.0396)	(0.00341)
Reference: No professional activity				
Part-time professional activity	-0.000819	-0.00724	-0.0478	-0.000444
	(0.0344)	(0.0576)	(0.0368)	(0.00226)
Regular professional activity	-0.0352	0.192	0.0538	0.00406
	(0.0523)	(0.129)	(0.0433)	(0.00393)
Time Dummies	Yes	Yes	Yes	Yes
Observations	7006	6806	6755	6507
Individuals	1182	1179	1169	1176
N. instruments	75	77	75	77
AR2	-0.146	1.188	0.343	0.268
	p= .884	p= .235	p=.0.732	p=.788
Hansen J	60.73	70.05	56.52	62.26
	p= .486	p=.253	p=0.639	p=.503

Standard errors in parentheses. * $p < 0.10$, ** $p < 0.05$, *** $p < 0.01$
